# How do hypertrophic cardiomyopathy mutations affect myocardial function in carriers with normal wall thickness? Assessment with cardiovascular magnetic resonance

**DOI:** 10.1186/1532-429X-12-13

**Published:** 2010-03-15

**Authors:** Tjeerd Germans, Iris K Rüssel, Marco JW Götte, Marieke D Spreeuwenberg, Pieter A Doevendans, Yigal M Pinto, Rob J van der Geest, Jolanda van der Velden, Arthur AM Wilde, Albert C van Rossum

**Affiliations:** 1 Department of Cardiology, VU University Medical Center, Amsterdam, the Netherlands; 2 Interuniversity Cardiology Institute of the Netherlands, Utrecht, the Netherlands; 3 Department of Cardiology, HagaZiekenhuis, the Hague, the Netherlands; 4 Department of Clinical Epidemiology and Biostatistics, VU University Medical Center, Amsterdam, the Netherlands; 5 Department of Cardiology, University Medical Center Utrecht, Utrecht, the Netherlands; 6 Department of Cardiology, Academic Medical Center Amsterdam, Amsterdam, the Netherlands; 7 Department of Radiology, Leiden University Medical Center, Leiden, the Netherlands; 8 Department of Physiology, VU University Medical Center, Amsterdam, the Netherlands

## Abstract

**Background:**

Clinical data on myocardial function in HCM mutation carriers (carriers) is sparse but suggests that subtle functional abnormalities can be measured with tissue Doppler imaging before the development of overt hypertrophy. We aimed to confirm the presence of functional abnormalities using cardiovascular magnetic resonance (CMR), and to investigate if sensitive functional assessment could be employed to identify carriers.

**Results:**

28 carriers and 28 controls were studied. Global left atrial (LA) and left ventricular (LV) dimensions, segmental peak systolic circumferential strain (SCS) and peak diastolic circumferential strain rate (DCSR), as well as the presence of late Gadolinium enhancement (LGE) were determined with CMR. Septal and lateral myocardial velocities were measured with echocardiographic tissue Doppler imaging. lv mass and volumes were comparable between groups. Maximal septal to lateral wall thickness ratio (SL ratio) was larger in carriers than in controls (1.3 ± 0.2 versus 1.1 ± 0.1, p < 0.001). Also, LA volumes were larger in carriers compared to controls (p < 0.05). Both peak SCS (p < 0.05) and peak DCSR (p < 0.01) were lower in carriers compared to controls, particularly in the basal lateral wall. Focal LGE was present in 2 carriers and not in controls. The combination of a SL ratio >1.2 and a peak DCSR <105%.s^-1 ^was present in 45% of carriers and in none of the controls, yielding a positive predictive value of 100%. Two carriers and 18 controls had a SL ratio < 1.2 and peak DCSR >105%.s^-1^, yielding a negative predictive value of 90%. With multivariate analysis, HCM mutation carriership was an independent determinant of reduced peak SCS and peak DCSR.

**Conclusions:**

HCM mutation carriership is an independent determinant of reduced peak SCS and peak DCSR when LV wall thickness is within normal limits, and is associated with increased LA volumes and SL ratio. Using SL ratio and peak DCSR has a high accuracy to identify carriers. However, since carriers also display structural abnormalities and focal LGE, we advocate to also evaluate morphology and presence of LGE when screening for carriers.

## Background

Hypertrophic cardiomyopathy (HCM) is a relatively common cardiomyopathy with an estimated prevalence of 1:500 in the general population [1]. The clinical course has a large inter- and intrafamilial heterogeneity, ranging from mild symptoms of heart failure late in life to the onset of sudden cardiac death at young age. Over 430 mutations in mainly sarcomeric genes have been identified to cause HCM. It has been suggested that the hypertrophy in HCM is a compensatory mechanism for mutant-induced myocardial dysfunction [2-4]. This hypothesis is supported by experimental data demonstrating that sarcomeric dysfunction precedes hypertrophy in HCM animal models [5-7].

Limited clinical data is available on regional myocardial function in human HCM mutation carriers (carriers) when wall thickness is still within normal limits. Several studies using tissue Doppler imaging with echocardiography demonstrated that predominantly diastolic myocardial velocities were reduced prior to the development of manifest hypertrophy [8,9]. However, evaluation of myocardial function with tissue Doppler imaging was not accurate enough to rule out HCM mutation carriership [8-10].

The high spatial and temporal resolution of cardiovascular magnetic resonance (CMR) and its well developed capabilities to assess morphology and intramural myocardial deformation, are of specific interest to evaluate the regional function of human carriers with normal wall thickness [11,12].

In this study, we investigate whether segmental systolic and diastolic myocardial dysfunction is detectable with CMR and can be used to identify carriership in carriers with normal left ventricular (LV) wall thickness when compared to controls.

## Methods

### Patient selection

Carriers were included in whom LV wall thickness was less than 10 mm measured by routine echocardiography within one year before entering the study [12]. All carriers were free of any other systemic or cardiac disease, which might attribute to the development of LV hypertrophy.

As a control group, age and gender matched non-smoking healthy volunteers were selected who had no medical history, no known familial cardiac disease, no obesity and normal blood pressure. Family members of the carriers without the pathogenic mutation, who met the inclusion criteria of healthy volunteers, were also included. Study participants had to be in sinus rhythm, and free from standard exclusion criteria for CMR. In all participants, standard physical examination, CMR and echocardiography were performed.

The study was approved by the institutional medical ethics committee and conducted according to the declaration of Helsinki. Written informed consent was obtained from all participants before entering the study.

### Cardiovascular Magnetic Resonance

CMR was performed on a 1.5-Tesla whole body scanner (Magnetom Sonata, Siemens, Erlangen, Germany), using a six-channel phased-array body coil. All cine studies were acquired in a single breath hold of 8-10 seconds during mild expiration.

#### Cine imaging

After survey scans, a retro-triggered, balanced, steady-state free precession gradient-echo sequence was used for cine imaging. A cine dataset fully covering the left atrium (LA) and LV was acquired as described previously [13]. Image parameters were: slice thickness 5 mm, slice gap 5 mm, temporal resolution <50 ms, repetition time 3.2 ms, echo time 1.54 ms, flip angle 60 degrees and a typical in-plane image resolution of 1.3 by 1.6 mm. The number of phases within the cardiac cycle was set at 20. A high temporal resolution (14 ms) 3-chamber cine was obtained to determine opening and closure times of aortic and mitral valves. Brachial blood pressure was non-invasively measured directly after acquisition of the high temporal resolution 3-chamber cine.

#### Myocardial tissue tagging

A multiple breath-hold, retrospective triggered balanced steady state free precession myocardial sinus tagging sequence was obtained using the linearly increasing start-up angle approach [11]. Three LV short axis planes were positioned at 25 percent, 50 percent and 75 percent of the distance between the mitral valve annulus and the endocardial border of the apex on an end-systolic LV 4-chamber view, avoiding inclusion of the LV outflow tract. Imaging time per slice was approximately 3-4 minutes. Image parameters were: 7 mm slice thickness, temporal resolution 14.1 ms, repetition time 4.7 ms, echo time 2.3 ms, flip angle 20 degrees, and in-plane image resolution of 1.2 by 3.8 mm, with 7 mm tag spacing.

#### Late gadolinium enhancement imaging

Late gadolinium enhancement (LGE) images were obtained 10-15 minutes after injection of 0.2 mmol·kg^-1 ^gadolinium-dtpa [14]. A single breath-hold, inversion recovery turbo Fast Low Angle Shot sequence was used. All LGE images were ecg-gated to end-diastole and planned at the same image positions as the long and short axis LV cines. Image parameters were: 5 mm slice thickness, repetition time 4.0 ms, echo time 4.4 ms, flip angle 25 degrees and typical in-plane image resolution 1.3 by 1.3 mm. Typical inversion recovery time was 250 to 300 ms.

### Post processing

Cine images where analyzed off-line, using MASS analysis software (Medis medical imaging systems, Leiden, The Netherlands) blinded for genotypes. From the LA cine dataset, LA volumes were calculated from manually drawn endocardial contours in every phase of the cardiac cycle, excluding the pulmonary veins and including the LA appendage, as described previously [13]. LA maximum volume, LA minimum volume, LA volume at diastasis and LA volume prior to atrial contraction were determined. From these volumes, LA reservoir volume, LA passive emptying volume, LA active emptying volume and LA ejection function were calculated as described previously [13].

On the LV cine data set, epi- and endocardial contours were manually drawn in end-diastole and end-systole. Global LV parameters, including LV end-diastolic (ED) volume, LV end-systolic volume, stroke volume, LV ejection fraction (lvef) and LV mass were determined. Also, LV mass to LV volume ratio in end-diastole was determined [13]. All volumes and mass were normalized to body surface area. Isovolumetric relaxation time (ivrt) was calculated by subtracting aortic valve closure time from mitral valve opening time, determined on the high temporal resolution 3-chamber view.

For segmental analysis, the basal, mid and apical LV cine slices best corresponding with the myocardial tissue tagging slices were selected. These three slices were subdivided into 16 segments - excluding the apex- according to the standardized myocardial segmentation for tomographic imaging of the heart [15]. Per LV segment, mean ED wall thickness and mean end-systolic wall thickness were measured. Wall thickening was calculated by dividing (end-systolic wall thickness - ED wall thickness) by ED wall thickness. In addition, maximal septal-to-lateral wall thickness ratio (SL ratio) was calculated by dividing maximal ED wall thickness of the septum by maximal lateral ED wall thickness, and ED wall radius was defined as the mean radial distance for the centre point of the LV slice to the endocardial border. This was semi-automatically calculated using MASS software.

Offline analysis of the myocardial tissue tagging images was performed with in-house developed software, programmed in a matlab 7.1(R14) environment (The MathWorks, Natick, Massachusetts, United States of America), as described previously [11]. Segmental circumferential strain was calculated from Lagrangian strain as a percent change in length of a small line segment in the circumferential direction. Since myocardial fibers of the mid LV wall are predominantly oriented circumferentially and lie within the short axis image plane, circumferential strain was calculated only from the mid 50 percent of the LV wall [16].

From these segmental circumferential strain datasets, the following parameters were determined: peak systolic circumferential strain (peak SCS) and peak diastolic circumferential strain rate (peak DCSR).

### Echocardiography

Echocardiography was performed using a General Electric Vivid-7 (GE Vingmed Ultrasound, Horten, Norway) ultrasound system. Echopac (GE, Horten, Norway) was used for offline analysis of recordings. Values of presented parameters are the means of 3 recorded measurements per parameter. The echocardiogram was performed immediately before or after the CMR study to minimize the effect of differences in physiological conditions of the participants between echocardiographic and CMR measurements.

*Colour tissue Doppler imaging *was performed on an apical 4-chamber view using a 2.5-MHz transducer and frame rates over 80/second. Systolic, early diastolic and late diastolic peak myocardial velocities of the septal and lateral mitral valve annulus corner were obtained by placing a 6-mm sample volume at the junction of the mitral annulus at septal and lateral myocardial wall. The angle of incidence between the scan lines and motion of the base of the heart was minimized.

### Statistical Analysis

All data are presented as mean ± standard deviation. The unpaired Student's t-test was used for comparison of normally distributed global LV and LA parameters between carriers and controls or a non-parametric Mann-Whitney U test when appropriate.

For segmental analysis, ED wall thickness, wall thickening, peak SCS and peak DCSR were compared between carriers and controls. While interdependency of segments was considered within slices and patients, multilevel analysis allowing random intercepts was performed. With multiple regression analysis, the effect of HCM carriership, age, gender, ED wall thickness and ED wall radius on wall thickening, peak SCS and peak DCSR was evaluated, correcting for interdependency of segments. Pearson's correlation coefficient was used to describe bivariate correlations between continuous variables. All regression models were evaluated for interaction of main effects. Evaluation of within slice differences of segmental parameters was performed with a one-way anova, using the Bonferroni post-hoc test for multiple comparisons. The Bland-Altman method for agreement analysis was used to evaluate the intra- and interobserver agreement of the strain data [17]. Also, the coefficient of variability was calculated by dividing the standard deviation of two measurements by their mean, as described previously [18]. mlwin 2.02 (Center of MultiLevel Modelling (Bristol, United Kingdom) was used for multilevel analysis and Statistical Package of Social Sciences (spss for windows 14.0, Chicago, Illinois, United States of America) for all other statistical analysis. We used receiver operator characteristics to generate cut-off values to optimize sensitivity and specificity to distinguish carriers from controls. Comparison between ROC curves was performed according to the method described by Hanley and McNeil using Analyse-it Clinical Laboratory 2.12 (Analyse-It Software, Ltd.) [19]. A two-sided p-value at the < 0.05 level was considered statistically significant.

## Results

In total, 28 carriers (11 males) from nine different families were included, of whom 22 (79%) had a myosin binding protein C3 founder mutation (MYBPC3) (2327insG) and 6 (21%) an α-tropomyosin mutation (Glu62Gln) [20,21]. Fifteen of the 28 age- and gender-matched controls were confirmed genotype negative family members of the carriers. All global LV and LA data and regional LV data were comparable between genotype negative controls and selected healthy volunteers. Systolic and diastolic blood pressures were lower in carriers compared to controls but within normal limits, see table 1. In total, data from 896 segments were obtained. All segmental data from the cine images were analyzable. Segmental strain data from 408 out of 448 (91.1%) segments could be used for analysis in carriers, and from 375 out of 448 (83.7%) in controls. The remaining segments were not analyzable due to inconsistency of repetitive breath holding. Tissue Doppler echocardiography was performed in 15 carriers and 12 controls.

**Table 1:** Baseline characteristics

	Carriers (*n *= 28)	Controls (*n *= 28)
Age (years)	38 ± 13.2	39 ± 12.3
Gender (male/female)	11/17	11/17
systolic BP (mmHg)	115 ± 12	124 ± 12*
Diastolic BP (mmHg)	66 ± 10	72 ± 7*
Heart rate (beats per minute)	63 ± 9	67 ± 9
Body mass index (kg·m^-2^)	22.9 ± 2.6	22.7 ± 2.6

Data are presented as mean ± S.D. BP = blood pressure, carriers = hypertrophic cardiomyopathy mutation carriers. * = p < 0.05

### Global LA and LV volumes and function

**Figure 1:**
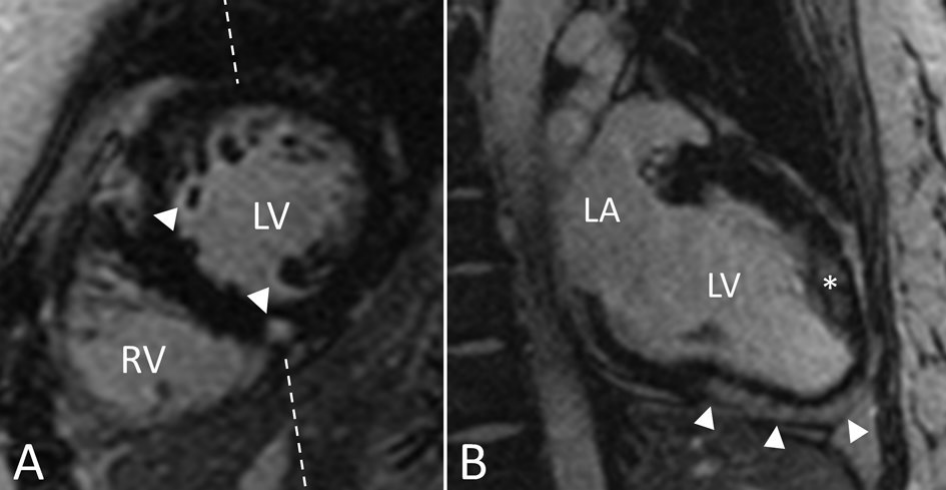
**LGE displaying a typical pattern of enhancement in a myosin binding protein C3 mutation carrier with normal LV wall thickness**. **A**. Midventricular LV short axis orientation on which enhancement is visible at the superior and inferior insertion point of the right ventricle into the septum (white arrowheads). These areas often typically display enhancement in manifest HCM patients. Image 1B was planned perpendicular to the inferior area of enhancement as indicated by the dashed line. **B**. Modified 2 chamber LGE image through the inferoseptum. The area of enhancement is indicated by the white arrowheads and is located in the midwall of the inferoseptum, extending towards the apex. * = anterolateral papillary muscle, LA = left atrium, LV = left ventricle, RV = right ventricle

Both LA minimum and LA maximum volume were larger in carriers compared to controls (p < 0.05) but LA passive emptying and LA active emptying were comparable, see table 2. No differences in global LV volumes, mass and isovolumetric relaxation time were found between both groups. In contrast, SL ratio was larger in carriers compared to controls (p < 0.001). LGE was observed in two MYBPC3 carriers (7%) and not in controls. Interestingly, the pattern of LGE was patchy and located typically at both insertion areas of the right ventricle into the non- hypertrophied septum, see figure 1.

**Table 2:** Left ventricular and left atrial volumes and function

	Carriers (*n *= 28)	Controls (*n *= 28)
LA min (mL.m^-2^)	24 ± 6.7	20 ± 3.9*
LA max (mL.m^-2^)	56 ± 11.7	51 ± 6.4*
LA PE (mL.m^-2^)	19 ± 4.6	18 ± 5.0
LA AE (mL.m^-2^)	14 ± 4.8	13 ± 3.1
LAEF (%)	35 ± 8.0	38 ± 7.2
LVEDV (mL.m^-2^)	96 ± 13.2	94 ± 14.2
LVESV (mL.m^-2^)	38 ± 7.3	37 ± 8.3
SV (mL.m^-2^)	58 ± 8.9	57 ± 7.7
LVEF (%)	60 ± 4.9	61 ± 4.2
LV mass (gr.m^-2^)	99 ± 24.2	93 ± 22.9
LV mass/volume ratio	0.53 ± 0.075	0.54 ± 0.105
IVRT (ms)	102 ± 18	100 ± 22
SL ratio	1.3 ± 0.21	1.1 ± 0.13^‡^

Data are presented as mean ± S.D. All volumes are indexed to body surface area, AE = active emptying, EDV = end diastolic volume, ESV = end systolic volume, IVRT = isovolumetric relaxation time, LA = left atrial, LAEF = left atrial ejection function, LV = left ventricular, LVEF = left ventricular ejection fraction, max = maximum volume, min = minimum volume, PE = passive emptying. SL ratio = septal to lateral wall thickness ratio, SV = stroke volume. * = p < 0.05, ‡ = p < 0.001

### Segmental analysis

All data per segment are presented in Additional file 1. The analysis of strain data had a high level of intra- and interobserver agreement for all strain parameters with a variability ranging from 1% for peak SCS, to 9% for peak DCSR, see table 3.

**Table 3:** Intra- and interobserver variability of strain parameters obtained with myocardial tissue tagging.

	intraobserver variability		interobserver variability	
	
	**mean difference ± S.D**.	coefficient of variation	**mean difference ± S.D**.	coefficient of variation
peak SCS (%)	0.03 ± 0.25	0.02	0.16 ± 1.3	0.01
peak DCSR (%·s^-1^)	0.02 ± 0.3	0.01	0.04 ± 9.5	0.09

S.D. = standard deviation, DCSR = diastolic circumferential strain rate, SCS = systolic circumferential strain

#### End diastolic wall thickness

**Figure 2:**
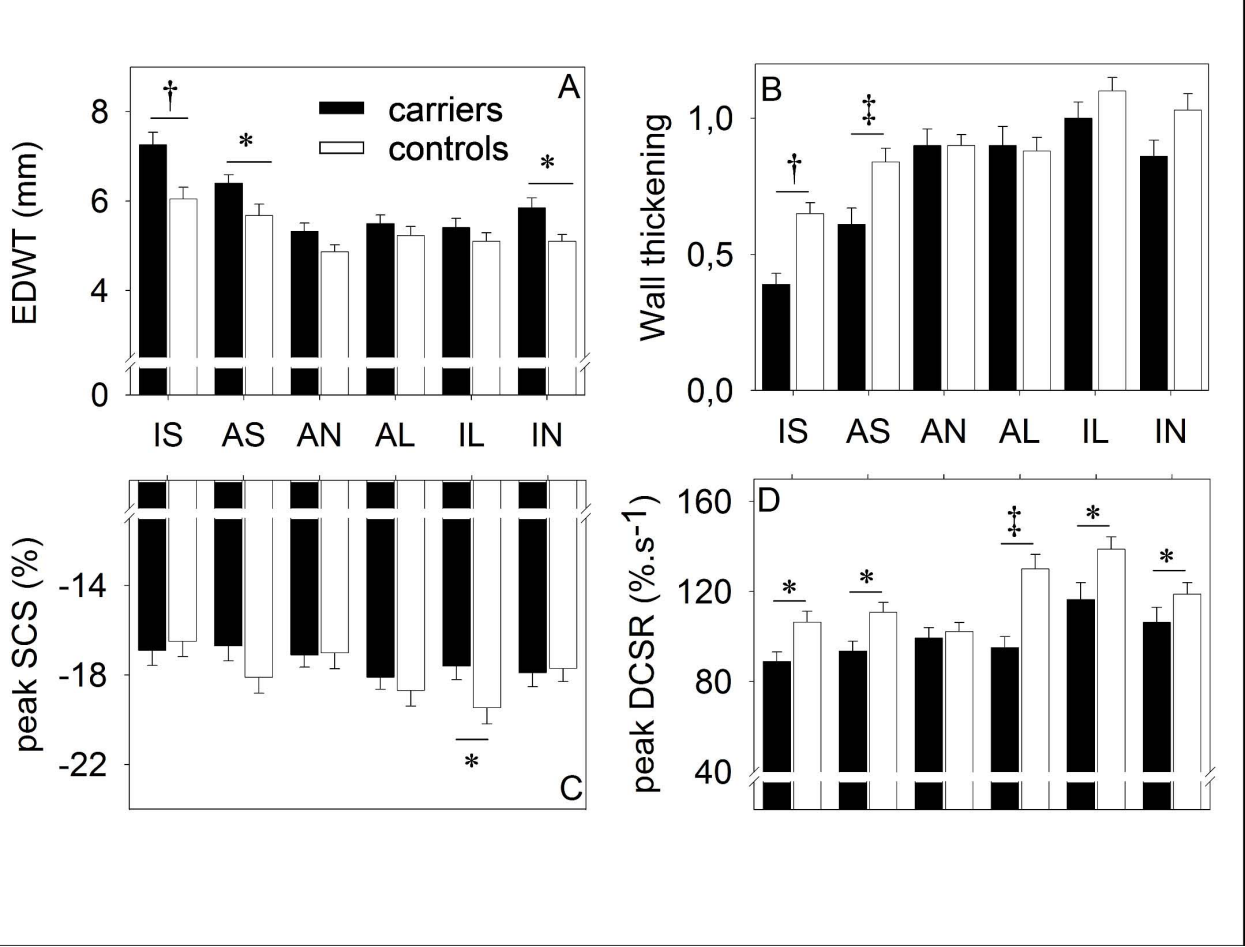
**Segmental comparison of basal left ventricular segments between carriers and controls**. Data are presented as mean ± standard error of the mean as indicated by the solid bars (carriers) and open bars (controls). **A**. EDWT was higher in the septal and inferior segments in carriers compared to controls. Also the asymmetric, predominantly septal distribution of increased wall thickness was observed in the carriers. **B**. Wall thickening was lower in the septum in carriers compared to controls. **C**. In controls, peak SCS was higher in the lateral wall compared to the septum, but this difference was less overt in carriers. As a result, peak SCS was significantly larger in the lateral segments of controls compared to carriers. **D**. Peak diastolic circumferential strain rate is reduced in almost every segment in carriers compared to controls. Again, the heterogeneity in peak DCSR found in controls was less profound in carriers. IS = inferoseptal, AS = anteroseptal, AN = anterior, AL = anterolateral, IL = inferolateral, IN = inferior, EDWT = end diastolic wall thickness, peak DCSR = peak diastolic circumferential strain rate, peak SCS = peak systolic circumferential strain. * = p < 0.05, ^† ^= p < 0.01, ^‡ ^= p < 0.001

Averaged ED wall thickness was comparable between carriers and controls (5.9 ± 0.9 mm versus 5.1 ± 0.9 mm in the basal slice, 4.9 ± 0.9 mm versus 4.7 ± 0.8 mm in the mid slice and 3.9 ± 1.0 mm versus 3.7 ± 0.8 mm in the apical slice, p = 0.23). In both septal segments of the basal slices, ED wall thickness was larger in carriers compared to controls (7.3 ± 1.5 mm versus 6.1 ± 1.4 mm, p < 0.01 for the inferoseptal segments and 6.4 ± 1.0 mm versus 5.7 ± 1.3 mm, p < 0.05 for the anteroseptal segments), see figure 2a. In all other segments, ED wall thickness was comparable (mean 4.9 ± 0.8 mm versus 5.0 ± 1.3 mm, p = 0.18).

#### Wall thickening

In both basal septal segments, wall thickening was lower in carriers compared to controls (0.39 ± 0.21 versus 0.65 ± 0.23, p < 0.001 for the inferoseptal segment and 0.61 ± 0.31 versus 0.84 ± 0.28, p < 0.01 for the anteroseptal segment). In the lateral segments, wall thickening was comparable between groups, see figure 2b.

#### Peak systolic circumferential strain

No significant differences in averaged peak SCS were observed between carriers and controls. Peak SCS was lower only in the basal inferolateral segment in HCM carriers compared to controls (-17.8 ± 3.3% versus -19.9 ± 3.5%, p < 0.05), see figure 2c. Interestingly, peak SCS was comparable between septal and lateral segments in carriers, but not in controls (p < 0.001), see figure 2c.

Multilevel regression analysis showed that HCM mutation carriership, (β = 0.66 ± 0.26, p < 0.001 indicating lower peak SCS in carriers), gender (β = -0.86 ± 0.26, p < 0.001 indicating larger peak SCS in females compared to males) and ED wall thickness (β = 0.39 ± 0.10, p < 0.01) were all independent predictors of peak SCS. There was no interaction of main effects. The effect of ED wall radius on peak SCS was significantly larger in controls (p < 0.05) compared to carriers; regression coefficients β were -0.19 ± 0.03 (p < 0.001) and -0.11 ± 0.04 (p < 0.01) respectively, indicating that an increase in ED wall radius was related to larger peak SCS. This effect was stronger in controls compared to carriers (p < 0.05).

#### Peak diastolic circumferential strain rate

Averaged peak DCSR was lower in all slices in carriers, especially in the basal slice (98 ± 18%·s^-1 ^versus 115 ± 17%·s^-1^, p < 0.001). In addition, the difference in peak DCSR between carriers and controls was largest in the basal lateral segments (95 ± 23%·s^-1 ^versus 130 ± 30%·s^-1^, p < 0.001 in the anterolateral segment and 116 ± 36%·s^-1 ^versus 139 ± 26%·s^-1^, p < 0.05 in the inferolateral segment), see figure 2d. In both carriers and controls, peak DCSR was highest in the inferolateral segments compared to the other segments (p < 0.01).

With multilevel regression analysis, HCM mutation carriership, (β = -9.0 ± 2.26; p < 0.001), gender (β = 11.4 ± 2.4; p < 0.001) and ED wall thickness (β = -3.5 ± 0.9; p < 0.001) were all independent determinants of peak DCSR. Again, ED wall radius was an independent determinant of peak DCSR in controls (p < 0.001), but not in carriers.

### The effect of ED wall thickness on regional function

**Figure 3:**
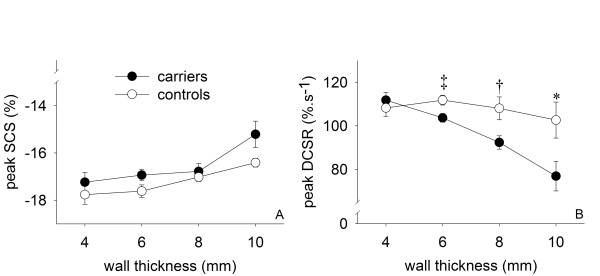
**Relation between functional parameters and different categories of EDWT**. Note that both peak SCS (**A**) and peak DCSR (**B**) tend to decrease with an increase in wall thickness in carriers (solid dots), but not in controls (open dots). EDWT = end diastolic wall thickness, peak DCSR = peak diastolic circumferential strain rate, peak SCS = peak systolic circumferential strain. * = p < 0.05, ^† ^= p < 0.01, ^‡ ^= p < 0.001

The effect of ED wall thickness on segmental functional parameters is illustrated in figure 3. Mean peak DCSR was significantly lower in carriers when ED wall thickness was ≥ 6 mm and further decreased with increase of ED wall thickness. Peak SCS only tended to be lower in carriers than in controls when ED wall thickness was >10 mm (-15.6 ± 3.5% versus -17.6 ± 1.6%, p = 0.05).

Septal and lateral systolic, early diastolic and late diastolic velocities assessed with tissue Doppler imaging were comparable between both groups in our study population, see table 4.

**Table 4:** Tissue Doppler Imaging parameters of HCM mutation carriers and controls

		Carriers (*n *= 16)	Controls (*n *= 12)
septal	Sa (cm·s^-1^)	8.7 ± 1.4	9.5 ± 2.4
	Ea (cm·s^-1^)	9.5 ± 3.2	9.5 ± 2.7
	Aa (cm·s^-1^)	8.0 ± 1.4	9.2 ± 2.2
lateral	Sa (cm·s^-1^)	10.8 ± 3.3	12.3 ± 2.5
	Ea (cm·s^-1^)	13.0 ± 4.5	12.7 ± 3.4
	Aa (cm·s^-1^)	9.0 ± 2.1	10.3 ± 2.6

Data are presented as mean ± standard deviation. Aa = late diastolic myocardial velocity at the level of the mitral valve annulus, Ea = early diastolic myocardial velocity at the level of the mitral valve annulus, Sa = systolic myocardial velocity at the level of the mitral valve annulus.

### Identification of carriers

**Figure 4:**
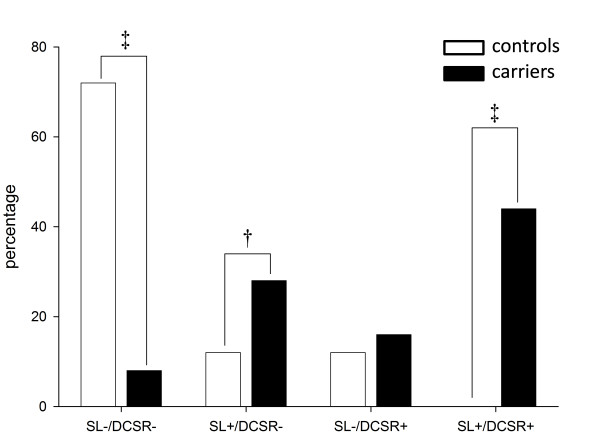
**Combining the evaluation of SL ratio and peak DCSR in the basal inferolateral segment from the identification of carriers**. Optimal cut-off was >1.2 and < 105%·s^-1 ^to positively identify carriers (solid bars). Note that only 2/25 (8%) of carriers did not meet neither this criteria, and no controls (open bars) met both criteria. Peak DCSR = peak diastolic circumferential strain rate, SL ratio = septal to lateral wall ratio. ^† ^= p < 0.01, ^‡ ^= p < 0.001.

Receiver operator characteristics analysis identified both SL ratio and peak DCSR of the lateral wall of the basal segment as valuable parameters to discriminate carriers from controls. Area under curve of SL ratio and basal inferolateral peak DCSR were comparable (0.72 ± 0.07, p < 0.001 and 0.69 ± 0.08, p = 0.02). Using an optimal cut-off value of 1.2 for SL ratio yielded a sensitivity, specificity, positive and negative predictive value of 75%, 88%, 81% and 77% respectively. An optimal cut-off value of 105%.s^-1 ^for peak DCSR within the lateral wall yielded a sensitivity, specificity, positive and negative predictive value was 58%, 80%, 73% and 65% respectively. As presented in figure 4, only 2 carriers (8%) had both an SL ratio <1.2 ánd a peak DCSR in the lateral wall >105%.s^-1 ^versus 18 (72%) of controls, p < 0.001, yielding a negative predictive value of 90%. Seven carriers (28%) had a SL ratio >1.2 and peak DCSR >105%.s^-1 ^versus 3 controls (7%, p < 0.01). In addition, 11 carriers (45 percent) had a SL ratio >1.2 ánd a peak DCSR <105%.s^-1 ^versus none of the controls, p < 0.001, yielding a positive predictive value of 100%. Thus, combining both functional and morphological parameters increases the accuracy to identify carriers, but does not completely exclude HCM mutation carriership.

## Discussion

This is the first study that used CMR to assess global LA and LV volumes and regional intramural myocardial function in carriers with normal wall thickness. The asymmetry in wall thickness between the septum and lateral wall, which is characteristic for HCM, was already present in these carriers with normal wall thickness. Typical focal LGE was present in 2 carriers. Also, LA volumes were larger in carriers. In addition, HCM mutation carriership was identified as an independent determinant of reduced peak SCS and peak DCSR, which was predominantly present in the basal lateral segments. Peak DCSR further deteriorated with increase in wall thickness. Using both the SL ratio and peak DCSR for identification of carriers had a high accuracy to identify carriers, but did not completely exclude HCM mutation carriership.

The finding of myocardial dysfunction in carriers with normal wall thickness supports the hypothesis that the hypertrophy in HCM represents a compensatory mechanism in response to abnormal intrinsic myocardial mechanical properties, as has been stated previously [2,8,9]. In a study performed by Ho and co-workers, who used tissue Doppler imaging to measure myocardial function in carriers with normal wall thickness, early diastolic velocities were found to be lower in carriers compared to controls in all corners of the mitral valve annulus [9]. This is in line with the findings in this study. However, myocardial velocities in that study were higher compared to this population, which might result from the younger age in their population (24.2 ± 10 years in the study of Ho and co-workers versus 38 ± 13 years in this study) [9]. The younger age and higher early diastolic velocities might also explain why, in contrast to our findings, LA volumes were not enlarged. Moreover, Ho and co-workers found that LVEF was significantly higher in carriers. This may again be an age-related effect, but may also be caused by differences in the measurement method of LVEF, since with echocardiography, papillary muscles are excluded from the LV mass, while we included the papillary muscles in the LV mass according to standardized CMR methods [22].

A study performed by Nagueh and co-workers, who also measured myocardial function with tissue Doppler imaging in a small cohort of HCM mutation carriers, found that both systolic and diastolic myocardial velocities were reduced [9]. However, wall thickness was higher in their study and most HCM mutation carriers in their population subsequently developed HCM within two years after inclusion into the study [23].

Therefore, diastolic dysfunction seems to be the earliest clinically detectable functional alteration as a result of the mutation. This is in line with experimental data on functioning of mutated sarcomeric proteins in a HCM model, which demonstrate that as a result of the HCM mutation, Ca^2+ ^sensitivity of the myofilaments is increased, thereby enhancing initial contractility but reducing myocardial relaxation [24-26]. After evaluation of all 16 segments, we found that reduced diastolic function in carriers was mainly caused by reduced peak DCSR in the basal segments of the myocardium and were most profound in the lateral segments.

In previous observations, we found in that peak SCS was largest in the inferolateral segments of the LV in healthy volunteers [11]. In this study, we found that ED wall radius was largest in the inferoseptal segments. Indeed, ED wall radius was found to be an independent determinant of peak SCS and peak DCSR in controls, suggesting a Frank-Straling mechanism. However, this relation was not found in HCM mutation carriers. This would explain why differences in peak SCS and peak DCSR found between controls and HCM mutation carriers were most profound in the inferolateral wall. Indeed an impaired response to preload in mutated sarcomeres has also been suggested in experimental studies [24]. However, this needs to be confirmed in further studies.

The reduction of regional peak DCSR was further enhanced with increase in ED wall thickness. It is unlikely that replacement fibrosis plays an important role in the deterioration of diastolic function in carriers with increase of ED wall thickness. However, diffuse interstitial fibrosis and/or myocyte disarray cannot be visualized with LGE imaging, so the attribution of these histological hallmarks of HCM on the development of diastolic dysfunction still needs to be further investigated. New promising CMR techniques have been developed that allow quantification of diffuse interstitial fibrosis. This would enable us to quantify the total fibrotic burden of the LV in HCM patients [27]. In this study, two MYBPC3 carriers demonstrated LGE, and the pattern of enhancement was typical for HCM, being located at the insertion sites of the right ventricle into the septum. LGE in carriers likely represents early myocardial damage that results from either micro infarction as a result of intramural coronary arteriopathy - which is often found in HCM - or concomitant myocardial inflammation [28]. Whether LGE in carriers heralds propagation to manifest HCM, or indicates an increased risk for ventricular arrhythmias needs to be clarified in future research.

### Clinical implications

Since HCM is relatively uncommon with an estimated prevalence in general population of 1:500, screening for carriers is only efficient in first degree, asymptomatic family members of HCM patients, who have a 50 percent pre-test likelihood of disease. In this preselected population, the presence of both a SL ratio >1.2 and a peak DCSR in the lateral segments <105%.s^-1 ^had a positive predictive value of 100% for carriership. Having a SL ratio <1.2 and a peak DCSR >105%.s^-1 ^had a negative predictive value of 90 percent. Therefore, functional abnormalities alone are not specific enough to be used to exclude HCM mutation carriership when screening asymptomatic family members of HCM patients in whom no mutation has yet been identified.

Since structural abnormalities, as well as focal areas of LGE have also been described in carriers with normal wall thickness, we advocate to also include evaluation of morphology and presence of LGE when screening for carriers to increase the accuracy of screening [12,29,30]. In addition, these findings might help to estimate the risk of carriers to develop HCM and/or arrhythmias.

### Limitations

Not all circumferential strain data were analysable, which may potentially have introduced a selection bias of the results. Also, baseline diastolic blood pressure was significantly lower in carriers, which may be related to the selection of carriers without LV hypertrophy. Study numbers are limited, which may have caused non-significant results due to insufficient power. Also, we only evaluated 2 mutations. Yet, these mutations in these genes account for approximately 25% of the total HCM population in the Netherlands, and about 20% of HCM patients worldwide [21]. The slice positions of the short axis SSFP cines did largely overlap the slice positions of the tagging slices, though did not completely correlate. Although unlikely, this methodological limitation may have affected the results. In this study, we evaluated only the accuracy of deformation in the circumferential direction in identifying carriers. The accuracy of deformation in radial and/or longitudinal direction for this purpose remains to be elucidated.

## Conclusions

HCM mutation carriership is an independent determinant of reduced peak SCS and peak DCSR when LV wall thickness is within normal limits, and is associated with increased LA volumes and SL ratio. Using SL ratio and peak DCSR has a high accuracy to identify carriers. However, since carriers also display structural abnormalities and focal LGE, we advocate to also evaluate morphology and presence of LGE when screening for carriers.

## Abbreviations

Carriers: hypertrophic cardiomyopathy mutation carriers; CMR: cardiovascular magnetic resonance; ED: end diastolic; HCM: hypertrophic cardiomyopathy; LA: left atrial; LGE: late gadolinium enhancement; LV: left ventricular; LVEF: left ventricular ejection fraction; MYBPC3: myosin binding protein C3; peak DCSR: peak diastolic circumferential strain rate; peak SCS: peak circumferential strain rate; SL ratio: maximal septal-to-lateral wall thickness ratio.

## Competing interests

The authors declare that they have no competing interests.

## Authors' contributions

TG - development of concept, gathering data, writing the manuscript. IKR - development of myocardial tagging quantification software, analysis of data, writing part of the methods section. MJWG - development of concept, writing part of the discussion section, critical revision of intellectual content. MDS - statistical analysis of data. PAD - development of concept, critical revision of intellectual content. RJvdG - development of software to measure en-diastolic wall radius, critical revision of intellectual content. YMP - development of concept, patient selection, critical revision of intellectual content. JvdV - development of concept, writing parts of the discussion section. AAMW - development of concept, patient selection, critical revision of intellectual content. ACvR - development of concept, chair of department, critical revision of intellectual content, final approval of manuscript submitted All authors read and approved the final manuscript.

## Supplementary Material

Additional file 1 **Appendix A**. segmental data of carriers and controls Click here for file
