# Supplementary material for: How do hypertrophic cardiomyopathy mutations affect myocardial function in carriers with normal wall thickness? Assessment with cardiovascular magnetic resonance
Source: J Cardiovasc Magn Reson. 2010 Mar 15;12(1):13. doi: 10.1186/1532-429X-12-13 (PMC2842263; doi:10.1186/1532-429X-12-13)
Supplement: Additional file 1 — Appendix A. segmental data of carriers and controls [file 1532-429X-12-13-S1.DOC]

Appendix A: segmental data of carriers and controls

|  | | ED wall thickness (mm) | | ED wall radius (mm) | | Wall thickening | | peak SCS (%) | | peak DCSR (%∙s^-1^) | |
| --- | --- | --- | --- | --- | --- | --- | --- | --- | --- | --- | --- |
|  |  | Carriers | Controls | Carriers | Controls | Carriers | Controls | Carriers | Controls | Carriers | Controls |
| basal slice | IS | 7.3±1.5 | 6.1±1.4 | 28±2.5 | 28±2.4 | 0.39±0.21 | 0.65±0.23 | -16.9±3.3 | -16.5±3.2 | 86±20 | 102±25 |
|  | AS | 6.4±1.0 | 5.7±1.3 | 27±2.4 | 27±2.7 | 0.61±0.31 | 0.84±0.28 | -16.8±3.2 | -18.1±3.3 | 93±21 | 109±31 |
|  | AN | 5.3±1.0 | 4.9±0.8 | 31±2.4 | 30±2.0 | 0.94±0.31 | 0.90±0.23 | -17.1±2.7 | -17.0±3.3 | 99±22 | 106±27 |
|  | AL | 5.5±1.2 | 5.2±1.0 | 27±2.1 | 28±2.2 | 0.95±0.38 | 0.88±0.27 | -18.1±2.6 | -18.7±3.4 | 95±23 | 130±30 |
|  | IL | 5.4±1.1 | 5.1±1.0 | 28±2.4 | 28±2.7 | 1.02±0.34 | 1.11±0.28 | -17.8±3.3 | -19.9±3.5 | 116±36 | 139±26 |
|  | IN | 5.8±1.5 | 5.1±0.8 | 30±2.3 | 30±2.2 | 0.86±0.31 | 1.03±0.30 | -17.8±2.9 | -17.8±2.8 | 106±33 | 127±27 |
|  | mean | 6.0±1.4 | 5.3±1.1 | 28±2.4 | 28±2.3 | 0.79±0.38 | 0.90±0.30 | -17.4±2.9 | -17.9±3.3 | 98±18 | 115±17 |
| mid slice | IS | 5.9±1.5 | 5.4±0.9 | 26±2.0 | 26±2.7 | 0.84±0.23 | 0.89±0.25 | -17.0±3.0 | -15.8±2.0 | 94±30 | 106±31 |
|  | AS | 5.0±0.9 | 5.0±0.9 | 27±2.0 | 26±2.7 | 0.88±0.23 | 0.85±0.25 | -16.4±3.0 | -17.3±2.9 | 104±28 | 108±31 |
|  | AN | 4.3±1.0 | 4.5±1.0 | 27±2.2 | 27±2.7 | 1.0±0.37 | 0.88±0.27 | -17.1±3.9 | -17.9±3.1 | 105±28 | 104±26 |
|  | AL | 4.3±1.0 | 4.7±1.0 | 26±2.0 | 26±2.8 | 1.11±0.43 | 0.90±0.30 | -17.2±3.1 | -18.9±3.5 | 98±27 | 114±37 |
|  | IL | 4.6±1.0 | 4.7±0.9 | 27±2.0 | 27±2.8 | 1.10±0.45 | 0.93±0.26 | -17.7±3.3 | -19.9±3.5 | 106±34 | 131±33 |
|  | IN | 5.1±1.5 | 4.9±0.7 | 27±2.0 | 27±2.7 | 1.0±0.32 | 0.88±0.25 | -16.2±3.0 | -16.9±2.9 | 98±31 | 115±39 |
|  | mean | 4.9±1.3 | 4.9±0.9 | 27±2.1 | 27±2.7 | 1.0±0.36 | 0.88±0.26 | -17.0±3.2 | -17.8±3.3 | 101±29 | 113±34 |
| apical slice | S | 4.0±1.0 | 4.4±0.9 | 20±2.7 | 18±3.5 | 0.98±0.36 | 0.90±0.32 | -15.3±4.0 | -14.3±3.1 | 107±32 | 87±36 |
|  | AN | 4.0±1.1 | 3.8±0.9 | 20±2.6 | 18±3.7 | 0.88±0.34 | 1.0±0.32 | -15.6±4.9 | -16.0±3.5 | 107±36 | 96±41 |
|  | L | 3.6±1.0 | 3.7±0.8 | 21±2.6 | 18±3.5 | 1.0±0.39 | 1.0±0.34 | -17.2±4.6 | -17.7±3.7 | 119±37 | 106±32 |
|  | IN | 3.8±1.1 | 3.8±0.9 | 20±2.6 | 18±3.6 | 0.98±0.34 | 0.94±0.36 | -15.6±4.4 | -15.2±3.4 | 109±32 | 112±42 |
|  | mean | 3.9±1.0 | 3.9±0.9 | 20±2.6 | 18±3.6 | 0.96±0.36 | 0.98±0.33 | -15.9±4.4 | -15.8±3.6 | 111±34 | 101±39 |

AL= anterolateral, AN= anterior, AS= anteroseptal, DCSR= diastolic circumferential strain rate, ED=end diastolic, IL= inferolateral, IN=inferior, IS=inferoseptal, L= lateral, SCS= systolic circumferential strain.
